# topPTM: a new module of dbPTM for identifying functional post-translational modifications in transmembrane proteins

**DOI:** 10.1093/nar/gkt1221

**Published:** 2013-12-02

**Authors:** Min-Gang Su, Kai-Yao Huang, Cheng-Tsung Lu, Hui-Ju Kao, Ya-Han Chang, Tzong-Yi Lee

**Affiliations:** ^1^Department of Computer Science and Engineering, Yuan Ze University, Chung-Li 320, Taiwan and ^2^Department of Computer Science and Engineering, Graduate Program in Biomedical Informatics, Yuan Ze University, Chung-Li 320, Taiwan

## Abstract

Transmembrane (TM) proteins have crucial roles in various cellular processes. The location of post-translational modifications (PTMs) on TM proteins is associated with their functional roles in various cellular processes. Given the importance of PTMs in the functioning of TM proteins, this study developed topPTM (available online at http://topPTM.cse.yzu.edu.tw), a new dbPTM module that provides a public resource for identifying the functional PTM sites on TM proteins with structural topology. Experimentally verified TM topology data were integrated from TMPad, TOPDB, PDBTM and OPM. In addition to the PTMs obtained from dbPTM, experimentally verified PTM sites were manually extracted from research articles by text mining. In an attempt to provide a full investigation of PTM sites on TM proteins, all UniProtKB protein entries containing annotations related to membrane localization and TM topology were considered potential TM proteins. Two effective tools were then used to annotate the structural topology of the potential TM proteins. The TM topology of TM proteins is represented by graphical visualization, as well as by the PTM sites. To delineate the structural correlation between the PTM sites and TM topologies, the tertiary structure of PTM sites on TM proteins was visualized by Jmol program. Given the support of research articles by manual curation and the investigation of domain–domain interactions in Protein Data Bank, 1347 PTM substrate sites are associated with protein–protein interactions for 773 TM proteins. The database content is regularly updated on publication of new data by continuous surveys of research articles and available resources.

## INTRODUCTION

Protein post-translational modification (PTM) involving addition of chemical groups is an extremely important biological mechanism that adjusts the physical and chemical properties, folding, conformation, stability and activity of proteins; thus, PTM alters protein function (1). High-throughput mass spectrometry (MS)-based proteomics has identified >200 different PTMs (2), including phosphorylation for signal transduction; acetylation and methylation of histone for gene regulation; attachment of hydrophobic groups for membrane localization; and glycosylation for changing protein half-life, targeting substrates and promotion of cell–cell and cell–matrix interactions (3). In addition, protein sumoylation and ubiquitination are a particular PTM involving the addition of other peptides, which plays various roles in cellular processes such as protein stability and degradation, transcriptional regulation, apoptosis, DNA repair and progression through the cell cycle (4–6). Given the accumulating site-specific PTM data obtained by MS/MS experiments, dbPTM (7) was developed to store verified PTMs from various PTM resources. Recently, an updated version of dbPTM was proposed as an informative resource for investigating substrate specificity and functional association of PTMs (8).

Proteomic analyses have shown that a transmembrane (TM) protein containing peptides that extend from one side of a membrane to the other side has crucial roles in various biological processes such as cell signaling, transport of molecules and ions, bioenergetics, cell recognition and cell–cell communication (9). A genome-wide study has reported that 20–30% of the proteins encoded by a typical genome are TM proteins (10). The TM proteins can be classified by structure as alpha-helical proteins and beta-barrel proteins. Alpha-helical TM proteins are a main class of membrane proteins; an estimated 27% of all human proteins are alpha-helical membrane proteins (11). Beta-barrel TM proteins, which are found in the outer membranes of Gram-negative bacteria, in the cell walls of Gram-positive bacteria and in the outer membranes of mitochondria and chloroplasts, participate in essential cellular functions by acting as porins, transporters, enzymes, virulence factors and receptors (12). Given the importance of TM proteins in cellular processes, several databases associated with TM proteins have been proposed, such as TMPDB (13), PDB_TM (14), OPM (15), TOPDB (16), TOPDOM (17) and TMPad (18). The TMBB-DB database recently proposed by Freeman and Wimley (19) would integrate experimental and predicted beta-barrel TM proteins.

The biological effects of PTMs on TM proteins include phosphorylation for signal transduction and ion transport; acetylation for structure stability; attachment of fatty acids for membrane anchoring and association; and glycosylation for substrates targeting, cell–cell interactions and virus infection (20,21). Although many databases have been developed for TM proteins or PTMs, no dedicated public resource is available for investigating the functions of PTMs on TM proteins. The importance of PTMs in the functioning of TM proteins motivated this study to develop topPTM, a new dbPTM module for identifying functional PTMs and structural topology of TM proteins. The TM proteins with experimentally confirmed TM topologies were collected from available databases and research articles. However, owing to the difficulties of experimentally obtaining high-quality structures, TM proteins are notably under-represented in Protein Data Bank (PDB). To provide a full investigation of TM proteins, UniProtKB (22) protein entries containing information regarding membrane localization and membrane topology are used for a thorough study of TM proteins because they are considered potential TM proteins in topPTM. Because topology information is often incomplete, the most probable topology for each topPTM protein given the experimental constraints was also calculated using two effective TM topology prediction algorithms. With the support of dbPTM, all the experimentally verified PTMs were then mapped to TM proteins of topPTM. The functional analysis of PTMs on TM proteins was simplified by graphically visualizing all PTM sites and TM topologies on TM proteins.

**Table 1.** Data statistics of the experimentally verified TM proteins in topPTM

Resource	Number of experimentally verified TM proteins		
	All	Alpha-helical	Beta-barrel
TMPad	379	379	0
OPM	651	1435	44
TOPDB	1479	667	91
PDB_TM	785	556	96
UniProtKB	4964	4920	139
Total	5394	4991	170

## MATERIALS AND METHODS

**Figure 1.**
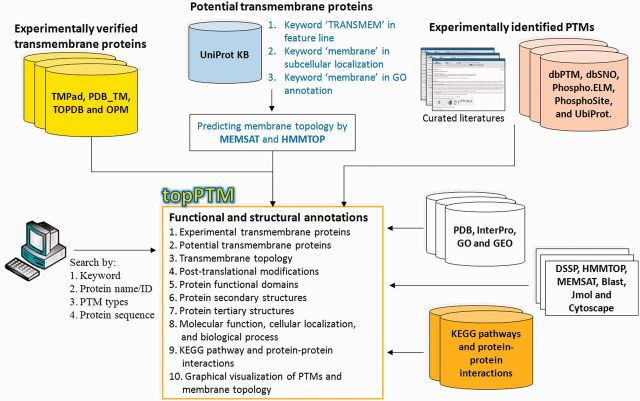
The system flowchart of topPTM construction.

Figure 1 depicts the system flowchart used to construct topPTM. Experimentally verified TM proteins annotated with membrane topology information were mainly collected from PDB_TM (14), OPM (15), TOPDB (16) and TMPad (18). After the removal of redundant protein entries, Table 1 shows that 5394 TM proteins containing experimentally curated annotations of TM topology remained. A set of candidate TM proteins was also extracted from UniProtKB by choosing protein entries containing the keyword ‘TRANSMEM’ in the feature (‘FT’) line, the localization of ‘membrane’ and the TM topology information. The candidate TM proteins were further filtered using HMMTOP (23) and MEMSAT (24) to determine their TM topologies. The filtering process obtained 69 402 potential TM proteins with annotated topologies.

In this work, the experimentally verified PTMs of TM proteins were mainly collected from dbPTM (7,8), which has integrated >10 public PTM resources. Given the emerging evidence of the effectiveness of MS/MS-based proteomics for identifying PTMs, site-specific modified peptides were also manually extracted from ∼500 MS/MS-associated research articles by applying a text mining approach (25). Deleting redundant PTM instances collected from various public resources then resulted in 4747 and 47 358 experimental PTM sites annotated on 1049 experimental and 8674 potential TM proteins, respectively. Statistical data for each PTM type shown in Supplementary Table S1 (Additional File 1) indicated that protein phosphorylation sites were the most common substrate sites in the experimental TM proteins and included 2108 phosphoserines on 603 TM proteins, 645 phosphothreonines on 333 TM proteins and 585 phosphotyrosines on 268 TM proteins. Additionally, 25 789 phosphoserines, 7510 phosphothreonines and 5939 phosphotyrosines were identified on potential TM proteins.

### Data integration for functional and structural investigations

For a given TM protein, basic data for biological functions were obtained from UniProtKB annotations. To obtain essential information about protein function and structure from annotations relevant to TM proteins, various biological databases, including Gene Ontology (GO) (26), InterPro (27) and PDB, were integrated. The preferences of biological functions for TM proteins were investigated by searching GO for annotations regarding molecular function, biological process and cellular component. InterPro is an integrated resource initially developed to rationalize the complementary efforts of the PROSITE (28), PRINTS (29), Pfam (30) and ProDom (31) databases to obtain protein ‘signatures’ such as protein families, domains and functional sites. The TM proteins play various roles in biological processes, including receptors for ligand binding, transporters for ions or molecules and starting points for signal transduction (32); most biological functions require interactions between TM proteins and other interacting partners (33). Protein interaction domains usually recognize short peptide motifs of a target protein but do not bind stably until the peptides have the appropriate PTMs; this can create binding sites for specific protein interaction domains that work together for cellular function and read the state of proteome to cellular organization (20). For instance, the CCR5 N-terminal domain peptide containing sulfotyrosines interacts consecutively with the human immunodeficiency virus type 1 (HIV-1) envelope glycoprotein gp120 to mediate the entry of certain HIV-1 strains into target cells (34). Thus, data for protein functional domains integrated with data for protein–protein interactions in dbPTM 3.0 enable inference of PTM-associated protein interactions. The topPTM provides a cross-link to dbPTM to enable access to information about PTM-associated protein interactions on TM proteins.

To provide a structural investigation of PTM sites on TM proteins, all of the experimentally verified PTM sites are mapped to the protein sequences of PDB using Blast (35) program. To support users investigating the PTM-associated interactions, the structural templates of domain–domain interactions in the PDB were extracted by referencing to the 3did (36), which provides molecular details for interaction interfaces. The substrate sites of PTMs locating in the interfaces of domain–domain interactions were regarded as the functional PTMs of TM proteins for PTM-associated interactions. Additionally, with an attempt to delineate the structural correlation between these annotated PTM sites and TM topologies, the tertiary structures were visualized using the Jmol package (37), and PTM substrate sites were highlighted on TM proteins.

### Database construction and availability

All data in topPTM are stored and managed using a relational MySQL database system. The web pages of the topPTM are implemented using PHP and JavaScript programming languages on Apache web server. To facilitate the study of PTMs on TM proteins, the structural topology and PTM sites of TM proteins were graphically represented using PHP GD library. The PDB tertiary structure of PTM sites on TM proteins was also visualized with Jmol package (37). The database content is maintained and updated quarterly by continuously surveying the public resources and research articles. The topPTM is now available at http://topPTM.cse.yzu.edu.tw. All experimentally verified PTMs and TM topologies on TM proteins could be downloaded in text format.

## DATA CONTENT AND UTILITY

### Structural distribution of PTMs on TM proteins with TM topologies

Table 2 presents the structural distribution of PTMs containing >10 substrate sites on experimental TM proteins according to the data for experimentally verified PTMs collected in the topPTM database. The structural topologies of a TM protein are mainly categorized into five types: Extracellular, Intracellular, TM, Other and Unknown regions. For instance, excluding substrate sites located in other and unknown regions, all N-linked glycosylation sites (2201 instances) and all substrate sites of O-linked and C-linked glycosylation are located in the extracellular region. Therefore, this study clarified the biological effect of glycosylation function on TM proteins during cell–cell interactions, cell recognition and virus infection (21). Analysis of structural distribution of protein phosphorylation showed that >80% of substrate sites (including 7857 phosphoserines, 2183 phosphothreonines and 2405 phosphotyrosines) are located in intracellular regions of TM proteins. This study also revealed the role of biological functions of protein phosphorylation in TM proteins that regulate intracellular signaling pathways. Several phosphorylation sites are also located in extracellular and TM regions of TM proteins. An emerging *S*-nitrosylation involved in the covalent attachment of nitric oxide to the thiol group of cysteine residues (38) is also known to locate preferentially in intracellular regions. Specifically, the N6-(retinylidene) lysine, which participates in light-driven ion transport and phototaxis signaling in microorganisms and has important roles in retinal isomerization and various types of photosignal transduction in higher animals (39), is located in TM regions. Supplementary Table S2 (in Additional File 1) shows the structural distribution of PTMs containing >10 substrate sites on all TM proteins in topPTM. Supplementary Figure S1 (in Additional File 1) shows that *Bacteriorhodopsin*, which consists of seven membrane-embedded alpha-helices that form an internal pocket in which the chromophore retinal is bound, contains an amino acid modification of N6-(retinylidene) lysine in TM region (40). The statistical analysis indicated that the structural distribution of PTMs is associated with their functional roles in TM proteins.

**Table 2.** The structural distribution of PTMs containing >10 substrate sites on experimental TM proteins

PTM Type	Number of substrate sites				
	Extracellular	Intracellular	Transmembrane	Other	Unknown
Phosphoserine	72	1603	24	210	199
Phosphothreonine	52	416	12	66	99
Phosphotyrosine	53	374	21	88	49
N-linked (GlcNAc … )	417	0	0	146	30
N6-acetyllysine	4	48	8	41	13
S-nitrosocysteine	8	26	6	12	18
N-linked (Glc … )	101	0	1	21	5
O-linked (GalNAc … )	57	0	0	6	0
S-cysteinyl 3-(oxidosulfanyl)alanine (Cys-Cys)	92	0	0	16	2
S-palmitoyl cysteine	0	32	4	1	6
N-acetylalanine	0	4	0	1	8
N-palmitoyl cysteine	0	17	1	0	2
N-myristoyl glycine	0	1	0	5	0
O-linked (GlcNAc)	3	4	0	0	1
N-acetylserine	0	12	0	4	4
N-acetylmethionine	1	5	1	1	4
S-farnesyl cysteine	0	0	0	0	0
Caspase cleavage aspartic acid	0	6	0	0	0
Methionine sulfone	0	4	0	0	0
N2,N2-dimethylarginine	1	4	0	4	0
N6-(retinylidene)lysine	0	0	57	0	0
5-methylarginine	1	3	0	4	0
S-geranylgeranyl cysteine	0	0	0	0	1
O-linked (GlcNAc … )	1	0	0	0	0
Cysteine methyl ester	0	0	0	0	1
4-hydroxyproline	0	2	0	0	0
O-linked (Man)	4	0	0	0	0
Asymmetric dimethylarginine	0	0	0	0	0
Pyrrolidone carboxylic acid	3	0	0	1	2
S-diacylglycerol cysteine	0	0	0	0	2
N-acetylthreonine	0	0	0	0	2
Sulfotyrosine	11	0	0	0	0
N-formylmethionine	0	2	1	5	4
N6,N6-dimethyllysine	0	0	0	0	0
Glutamate methyl ester (Glu)	0	2	0	0	0
Nitrated	0	3	1	0	1
Omega-N-methylarginine	0	0	0	0	3
Deamidated asparagine	0	1	0	0	0
O-linked (Man … )	0	0	0	0	0
N4-methylasparagine	0	0	0	0	0
(3S)-3-hydroxyasparagine	0	0	0	0	0
GPI-anchor amidated serine	1	0	0	0	1
N6-methyllysine	0	2	0	0	0
Omega-N-methylated arginine	0	3	0	0	0
Glutamate methyl ester (Gln)	0	2	0	0	0
N6-succinyllysine	0	0	0	0	0
N-linked (Glc)	0	0	0	0	0
C-linked (Man)	2	0	0	0	0
Phosphohistidine	0	1	0	0	1
Citrulline	0	0	0	0	0
Deamidated glutamine	0	0	0	0	0
Nitrated tyrosine	0	2	0	0	2
O-linked (Xyl … )	3	0	0	0	0
O-linked (Xyl … ) (glycosaminoglycan)	0	0	0	1	0
N-acetylglycine	0	0	0	0	0

### Web interface of topPTM

**Figure 2.**
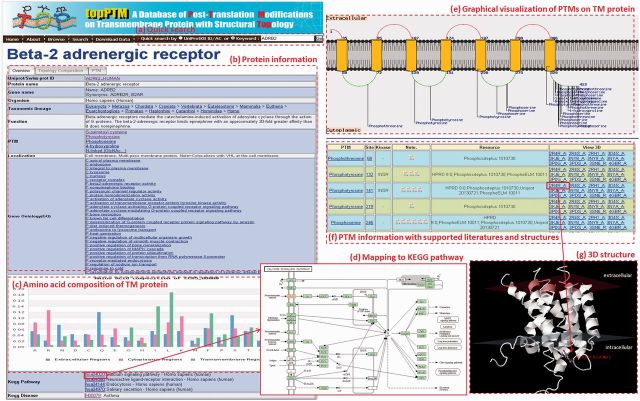
The web interface of a typical topPTM query. (**a**) Quick search by UniProtKB ID/AC or keywords. (**b**) Protein information. (**c**) Amino acid composition in extracellular, TM and intracellular regions of a TM protein. (**d**) Pathway analysis by mapping the TM protein to KEGG metabolic pathways. (**e**) Graphical visualization of PTM sites on TM protein with structural topology. (**f**) Detailed information of PTMs with supported literatures and 3D structures. (**g**) Jmol visualization of a specific PTM site on tertiary structure.

To facilitate access to topPTM resource, a web interface has been developed to enable efficient browsing and searching for TM proteins of interest. A typical topPTM query includes basic protein information, amino acid composition in different membrane topologies, pathway analysis, graphical visualization of PTMs on a TM protein with membrane topology, detailed information of PTMs with supported literatures and visualization of PTM sites on a tertiary structure by Jmol program. Figure 2 shows that, after users input a UniProtKB ID/AC or keyword into the ‘Quick search’ function, the topPTM efficiently returns a graphical visualization of PTMs and membrane topology and also provides basic information about the TM protein of interest. Additionally, amino acid compositions in extracellular, transmembrane and intracellular regions of TM proteins are represented in a bar chart. One purpose of topPTM is enabling functional analysis of PTMs on TM proteins. Thus, users can refer to a table containing detailed PTM data to determine the biological function of each modification site by referencing the supported literature. By viewing 3D structures with Jmol program, users can also investigate the structural environment of PTM substrate sites. Furthermore, the membrane boundaries, represented as planes in Jmol viewer, could help users to identify the structural distribution of PTM substrate sites on a TM protein.

### Functional investigation of PTMs on transmembrane protein

The statistical analyses of PTM distribution on TM proteins indicated that some PTMs have structural preferences for locating in extracellular, transmembrane or intracellular regions. For instance, protein phosphorylation preferentially locates in intracellular regions of TM proteins, whereas protein glycosylation preferentially locates in extracellular region of TM proteins. However, the location of PTMs on TM proteins is associated with their functional roles in various cellular processes (41). To identify the functional roles of PTMs on TM proteins, a text mining system that uses information retrieval technologies (42) has been developed for topPTM to extract research articles related to PTM function. Of the 4747 experimental PTMs, 2656 PTM substrate sites are located in the regions of functional domains of 1528 TM proteins. Given the support of research articles by manual curation and the investigation of domain–domain interactions in PDB, 1347 PTM substrate sites are associated with protein–protein interactions for 773 TM proteins.

**Figure 3.**
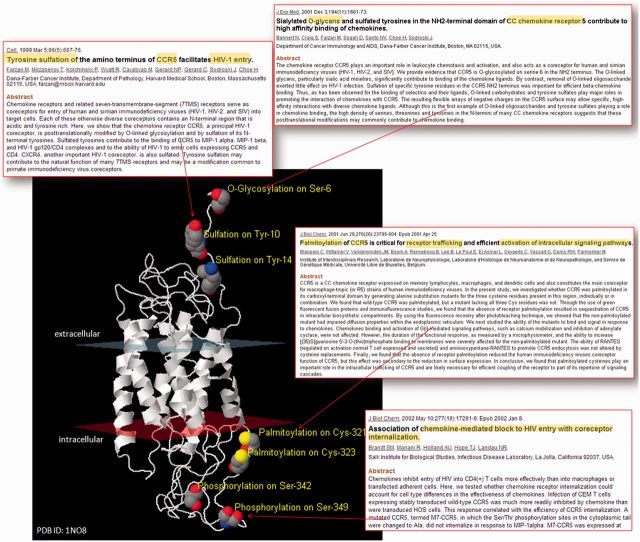
A case study of investigating the functions of PTMs on protein structure (PDB ID:1NO8) of human C–C chemokine receptor type 5 (CCR5).

Figure 3 shows the case study, in which 16 PTMs were experimentally determined on *C-C chemokine receptor type 5* (CCR5) in the family of human G-protein-coupled receptor 1. The graphical representation of PTMs and transmembrane topology of CCR5 provided by topPTM indicate that the sulfated tyrosines (occurring on positions 3, 10, 14 and 15) (43,44) and *O*-glycosylated serine (occurring on position 6) (45) are located in the extracellular region of the CCR5 N-terminus. Additionally, the phosphoserines (occurring on positions 336, 337, 342 and 342) (46–49), phosphotyrosine (occurring on position 339) (50), *S*-palmitoylated and *N*-palmitoylated cysteines (occurring on positions 321, 323 and 324) (51,52) are located in the intracellular region of CCR5 C-terminus. To enable structural analysis of PTM sites, the topPTM highlights the residues containing the annotation of PTMs on tertiary structures. As the tertiary structure of CCR5 (PDB ID: 1NO8) presented in Figure 3, the modified residues, such as Ser-6 of *O*-glycosylation, Tyr-10 and Tyr-14 of sulfation, Cys-321 and Cys-323 of palmitoylation and Ser-342 as well as Ser-349 of phosphorylation, are displayed as ‘space filling’ model in Jmol viewer. The gray and red planes stand for extracellular and intracellular membrane boundaries, respectively. Therefore, the structural distribution of PTM sites in CCR5 can be physically investigated.

Regarding the functional analysis of PTMs on CCR5, the topPTM gives a literature list containing the title and abstract of research articles associated with a specific PTM. The chemokine receptor CCR5 is known to have a crucial role in leukocyte chemotaxis and activation, and also acts as a co-receptor for HIVs (45). For the functional analysis of sulfotyrosine on CCR5, the interaction between CCR5 and the HIV-1 gp120/CD4 complex requires sulfation of two to four tyrosine residues in CCR N-terminus (44), which facilitates HIV-1 entry into target cells (43). Regarding the O-linked glycosylation, O-linked glycans located in the extracellular region of CCR5 are major contributors to the binding of the chemokine ligands; in contrast, removal of O-linked oligosaccharide has a minimal effect on HIV-1 infection (45). On the other hand, CCR5 phosphorylation and palmitoylation are known to have important roles in intracellular processes. The serine residues at positions 336, 337, 342 and 349 of CCR5 are phosphorylated by beta-adrenergic receptor kinase 2 (ADRBK2) (46), which is in the G protein-coupled receptor kinase (GRK) family. The CCR5 mutants that lack any two phosphorylation sites retain their ability to recruit endogenous beta-arrestins to the cell membrane and are normally sequestered, whereas alanine mutation of any three C-terminal serine residues abolishes both beta-arrestin binding and rapid agonist-induced internalization (48). With regard to the phosphotyrosine, vaccinia virus activation of CCR5 invokes tyrosine phosphorylation signaling events that support virus replication (50). Palmitoylated cysteines are reportedly critical in the intracellular trafficking of CCR5 and are likely necessary for efficient coupling of the receptor to part of its repertoire of signaling cascades (51).

## CONCLUSION

Owing to the importance of PTMs functioning on TM proteins, a new dbPTM module called topPTM was developed to identify the functional PTM sites on TM proteins with structural topology. The topPTM is the first public resource to enable efficient access to curated PTM sites, functional annotations, tertiary structures, membrane topologies and network contexts for transmembrane proteins. The case study of CCR5 in this work showed that topPTM is an informative system for providing structural distribution and functional investigation of PTMs for TM proteins. Further growth of topPTM is expected as the availability of data increases in resources related to PTMs and membrane topology. To provide the data needed for functional analysis, the descriptions associated with the biological function of PTMs will be extracted more precisely from research articles using an enhanced information retrieval system. Additionally, a previous work has reported that the second extracellular loop and amino-terminal domain of CCR5 are critical for chemokine binding, whereas the transmembrane helix bundle is involved in receptor activation (53). A previous study has demonstrated the consideration of transmembrane topology could decrease the false positives when predicting O-linked glycosylation sites on TM proteins (54). Thus, future works can also investigate the potential use of topPTM for identifying the substrate sites of PTMs on transmembrane proteins.

## SUPPLEMENTARY DATA

Supplementary Data are available at NAR Online.

## FUNDING

National Science Council of the Republic of China for financially supporting this research under Contract Numbers of [NSC 101-2628-E-155-002-MY2]. Funding for open access charge: National Science Council of Taiwan.

*Conflict of interest statement*. None declared.
